# Autologous stem cell transplantation as consolidation therapy for patients with peripheral T cell lymphoma in first remission: long-term outcome and risk factors analysis

**DOI:** 10.1007/s00277-013-1716-2

**Published:** 2013-03-08

**Authors:** Anna Czyz, Joanna Romejko-Jarosinska, Grzegorz Helbig, Wanda Knopinska-Posluszny, Lidia Poplawska, Beata Piatkowska-Jakubas, Dorota Hawrylecka, Barbara Nasilowska-Adamska, Dominik Dytfeld, Anna Lojko-Dankowska, Anna Kopinska, Piotr Boguradzki, Jan Walewski, Slawomira Kyrcz-Krzemien, Andrzej Hellmann, Mieczyslaw Komarnicki

**Affiliations:** 1 Department of Hematology, Poznan University of Medical Sciences, Szamarzewskiego 84, 61-569 Poznan, Poland; 2 Department of Lymphoid Malignancies, Maria Sklodowska-Curie Institute and Oncology Centre, Roentgena 5, 02-781 Warsaw, Poland; 3 Department of Hematology and Bone Marrow Transplantation, Silesian Medical University, Dabrowskiego 25, 40-032 Katowice, Poland; 4 Department of Hematology, Medical University of Gdansk, Debinki 7, 80-952 Gdansk, Poland; 5 Department of Hematology, Jagiellonian University Medical College, Kopernika 17, 31-501 Cracow, Poland; 6 Institute of Hematology and Blood Transfusion, Bone Marrow Transplantation Unit, Indiry Gandhi 14, 02-776 Warsaw, Poland; 7 Department of Hematology, Oncology and Internal Diseases, The Medical University of Warsaw, Banacha 1a, Warsaw, Poland; 8 Department of Hematology, Independent Public Hospital of Ministry of the Interior with Warmia and Mazury Oncology Centre, Wojska Polskiego 37, 10-228 Olsztyn, Poland; 9 Department of Hematology, Podkarpacie Oncology Centre, Ks. J.Bielawskiego 18, 36-200 Brzozow, Poland

**Keywords:** Peripheral T cell lymphoma, Autologous hematopoietic stem cell transplantation, Prognostic factors, Clinical outcomes, Scoring systems

## Abstract

This report is a retrospective analysis of 65 patients with peripheral T cell lymphoma (PTCL), who underwent high-dose therapy and autologous hematopoietic stem cell transplantation (autoHCT) as a consolidation of first response achieved with either induction or salvage chemotherapy. We intended to determine the prognostic factors that influenced outcome after autoHCT and to define the predictive value of the scoring systems most often applied for transplant outcomes. Nineteen patients in either complete or partial remission underwent autoHCT after induction chemotherapy. Forty-six patients received second-line chemotherapy as a consolidation of partial response after induction chemotherapy (*n* = 34) or as a salvage therapy after primary induction failure (*n* = 12), and thereafter proceeded to autoHCT. Finally, the 36 patients were in complete remission, and 29 in partial remission at autoHCT. The median follow-up of survivors was 53 months (range 7–157 months). The 5-year overall survival and progression-free survival for all patients were 61.5 % (95 % CI 47.0–74.2 %) and 59.4 % (95 % CI 46.1–71.5 %), respectively. In multivariate analysis, bone marrow involvement at diagnosis and less than partial remission after induction chemotherapy were factors independently predictive for overall survival and progression-free survival. The prognostic index for PTCL could reliably stratify the prognosis of PTCL in this analysis.

## Introduction

Although peripheral T cell lymphomas (PTCL) are relatively uncommon disorders, representing only 8–15 % of all non-Hodgkin lymphomas, some estimates have been reported indicating that the incidence of PTCL has been increasing for the last two decades, faster that the incidence of B cell lymphomas [1, 2]. The nodal types of PTCL, which include peripheral T cell lymphoma not otherwise specified (PTCL-NOS), anaplastic large cell lymphomas (ALCLs), and angioimmunoblastic T cell lymphoma, are a heterogenous group of diseases that are challenging to treat. Most PTCL subtypes have a poor prognosis with a 5-year survival rate of approximately 30 % and a median survival of 2–3 years [3, 4]. The exception is patients with anaplastic lymphoma kinase (ALK)-positive ALCL, who have a 5-year survival rate of about 60–90 % with conventional chemotherapy [3, 5]. Although the outcome of patients with PTCL is worse than that of patients with aggressive B cell lymphomas [6, 7], they are treated similarly with the CHOP or CHOP-like regimens used as an induction chemotherapy. Unfortunately, even up to 40 % of patients experience primary induction failure or early relapse after CHOP-like chemotherapy [4, 8, 9]. Moreover, the best treatment option for patients with PTCL who have responded to the conventional chemotherapy remains undefined. Several prospective phase II trials, as well as the results of retrospective studies, support high-dose therapy (HDT) followed by autologous hematopoietic stem cell transplantation (autoHCT) as a consolidation of first response for PTCL. Although autoHCT seems to extend progression-free survival (PFS) and overall survival (OS), unfortunately 20–40 % of patients will relapse following autotransplant [8, 10–12]. Since relapse remains the primary cause of autoHCT failure, allogeneic HCT after reduced intensity conditioning (RIC) regimen is sometimes offered to the selected patients in first remission, primary with high-risk histological subtypes of PTCL [13–15]. In addition, a variety of new drugs registered in relapsed disease are being studied in the upfront setting [16]. Hence, better characterization of prognostic factors, as well as validation of prognostic scores used in the transplant setting, is required for improved patient selection for autoHCT. The scoring systems most often applied in the literature of HCT for PTCL are the International Prognostic Index (IPI) and the Prognostic Index for Peripheral T cell Lymphoma not otherwise specified (PIT). The latter index is based on age, performance status, lactate dehydrogenase (LDH), and bone marrow involvement [17]. The usefulness of the IPI has been questioned in some studies in the autotransplant setting [8, 18]. In contrast, the PIT has been reported to be more accurate in stratifying PTCL patients undergoing autoHCT [8, 18, 19].

Since the results reported by several groups around the world suggested that HDT and autoHCT was beneficial in PTCL in the front-line setting, patients in first remission have been considered for autotransplant at our centers for more than decade now. To expand the published experience, we conducted a multicenter, retrospective review of patients with PTCL who underwent HDT and autoHCT as a consolidation of first response achieved with either initial induction chemotherapy or salvage chemotherapy. We intended to determine the overall survival, the progression-free survival, and the prognostic factors that influenced outcome after autoHCT. We also intended to define the predictive value of IPI and PIT scores for transplant outcomes of patients with PTCL in first remission. Herein, we report the results of this analysis.

### Patient selection

The records of all patients with a confirmed diagnosis of peripheral T cell lymphoma receiving HDT and autoHCT between 1998 and 2011 at each of the seven centers participating in the present retrospective analysis were reviewed. Included in the study were patients who received autoHCT in first response achieved with either induction or salvage chemotherapy. All the patients with primary cutaneous lymphoma were excluded from the analysis. The patients with ALK-positive ALCL and ALK-unknown ALCL who received autoHCT as a consolidation of first complete response achieved with the initial induction chemotherapy were also excluded from the study. In contrast, patients with ALK-positive and ALK-unknown ALCL who had achieved less than complete response after induction chemotherapy and received afterwards HDT and autoHCT, either preceded or not by second-line chemotherapy, were included in the analysis.

### Data collection and definitions

Patients records were reviewed to obtain baseline characteristic at the time of diagnosis (clinical stage, presence of B symptoms, performance status, bone marrow involvement, involvement of extranodal sites, mediastinal lymph node involvement, LDH, IPI score, PIT score). Complete response (CR), partial response (PR), and disease progression were defined using standard criteria [20]. Primary induction failure (PIF) was defined as the achievement less than partial response after induction chemotherapy.

### Statistical analysis

The endpoints were overall and progression-free survival. Survival curves were estimated according to the method of Kaplan and Meier. OS was measured from the time of transplantation until death from any cause and PFS was measured from the time of transplantation until documented progression or relapse or death from any reason.

The two-tailed logrank test was utilized to compare the curves. *p* values <0.05 were considered significant. Potential prognostic factors, histology, clinical stage, presence of B symptoms, bone marrow involvement, extranodal sites, mediastinal involvement, LDH, performance status, response to initial induction chemotherapy, a total number of chemotherapy lines before autoHCT, and disease status at transplant were evaluated for OS and PFS in univariate analysis. Cox proportional hazards model was used for multivariable analysis.

The predictive value of the IPI and PIT scoring systems for transplant outcome survival probabilities were estimated using Kaplan and Meier method. The logrank test was used to compare survival curves. HRs and 95 % confidential intervals (95 % CI) were determined using Cox regression method.

SPSS version 14.0 (SPSS, Chicago, IL) was used for all statistical analyses.

## Results

### Patient characteristics, prior treatment, and transplantation procedures details

From January 1998 to December 2011, the 65 patients (32 men and 33 women) received HDT and autoHCT as a consolidation of first response achieved with either induction or salvage chemotherapy. The median age at transplant was 42 years (range 15–64 years). Patient baseline characteristics are presented in Table 1. Fifty nine of the 65 patients (91 %) had received CHOP or CHOP-like regimen as an induction chemotherapy. Twelve patients in complete response and seven patients in partial response proceeded to autoHCT after induction chemotherapy. Thirty-four of the 65 patients (52 %) received second-line chemotherapy as a consolidation of partial response achieved with the induction chemotherapy and thereafter proceeded to autoHCT. The decision about consolidation of partial response with second-line chemotherapy was taken by the responsible physician. The decision depended on the tumor burden after induction chemotherapy and the practice of institutions involved in the study. Patients in partial response were considered to receive second-line chemotherapy at five of the seven institutions participating in this study. Twelve of the 65 patients (18 %) received second-line chemotherapy as a salvage treatment after primary induction failure. Having achieved at least a partial remission after salvage treatment, they underwent autoHCT. The patients received a median of two (range 1–4) chemotherapy lines. Finally, 36 patients were in CR and 29 in PR at autoHCT, respectively. The autologous graft source was mobilized peripheral blood in 95 % of all cases. The median number of infused CD34 positive cells was 5.6 × 10^6^ cells/kg (range, 1.6–22.8). Institutional transplant guidelines for supportive care were followed. Antiemetics prophylaxis was based on ondansetron. Antifungal, antiviral, and antibacterial prophylaxis included fluconazole, acyclovir, and ciprofloxacin or norfloxacin until neutrophil recovery. Packed red blood cells were administered to maintain Hb levels ≥4.8 mmol/L. Platelet transfusions were administered to keep platelet count ≥10 or ≥ 20 G/L in patients with increased risk of bleeding complications. Engraftment was documented in all but one patient who died within 10 days of transplant from infection. Recovery to granulocyte count >0.5 G/l occurred at a median 13 days (range, 10–18 days). Table 2 shows chemotherapy and transplant details.

**Table 1:** Baseline patient and disease characteristics

Characteristics at diagnosis	Number (%)
Total number of pts	65 (100)
Age (years), median 42, range 15–64	
≤60 years	61 (94)
>60 years	4 (6)
Gender	
Male	32 (49)
Female	33 (51)
Histology	
PTCL not otherwise specified	36 (55)
AITL	9 (14)
ALCL	20 (31)
ALK-negative	7 (11)
ALK-positive	4 (6)
ALK-unknown	9 (14)
Ann Arbor stage	
I–II	14 (21.5)
III–IV	50 (77)
Unknown	1 (1.5)
Constitutional symptoms	
Absent	14 (21.5)
Present	51 (78.5)
BM involvement	
No	44 (68)
Yes	16 (24.5)
Unknown	5 (7.5)
IPI score	
0–1	16 (25)
2	17 (26)
3–4	24 (37)
Unknown	8 (12)
PIT score	
0	10 (15)
1	23 (35)
2–3	18 (28)
Unknown	14 (22)

*PTCL* peripheral T cell lymphoma, *IPI* International Prognostic Index, *PIT* Prognostic Index for peripheral T cell lymphoma, *AITL* angioimmunoblastic T cell lymphoma, *ALCL* anaplastic large cell lymphoma, *ALK* anaplastic lymphoma kinase

**Table 2:** Previous treatment and transplant details

Treatment details	Number (%)
Induction chemotherapy	
CHOP	57 (88)
CHOP and etoposide	2 (3)
Others anthracycline-containing combination chemotherapies	6 (9)
Response after induction chemotherapy	
CR	12 (18)
PR	41 (64)
Primary refractoriness^a^	12 (18)
Second-line chemotherapy	46 (71)
ESHAP or DHAP	27 (42)
Other platinum-containing regimen	4 (6)
Others	15 (23)
Number of pre-transplant regimens	
1	18 (28)
2	42 (65)
>2	5 (7)
Disease status at autoHSCT	
CR	36 (55)
PR	29 (45)
Autologous graft source	
Mobilized peripheral blood	62 (95)
Bone marrow	3 (5)
The number of infused CD34 positive cells ×10^6^/kg	
Median	5.6
Range	1.6–22.8
Conditioning regimen	
BEAM	38 (58)
CBV	18 (28)
CyTBI	3 (5)
Others	6 (9)

*CR* complete response, *PR* partial response

^a^Less than PR

### Posttransplantation outcomes

All patients were evaluated for survival, with a median follow-up time of surviving patients of 53 months (range 7–157 months). A detailed analysis was carried out at the censor date (30 April 2012). Twenty-one (32 %) patients in our study have died. The cause of death in 17 patients was progressive disease. Four patients have died from non-relapse causes, corresponding to non-relapse mortality of 6 %. Two of the four patients have died as a result of infections, one patient died from heart failure 12 years after autoHCT, and one patient died 55 months after transplant as a consequence of a post-transplant diagnosed glioblastoma. With regard to secondary hematologic malignancies, one acute myeloid leukemia was observed 4 years after autoHCT that was successfully treated with chemotherapy at the time of the censor date.

**Fig. 1:**
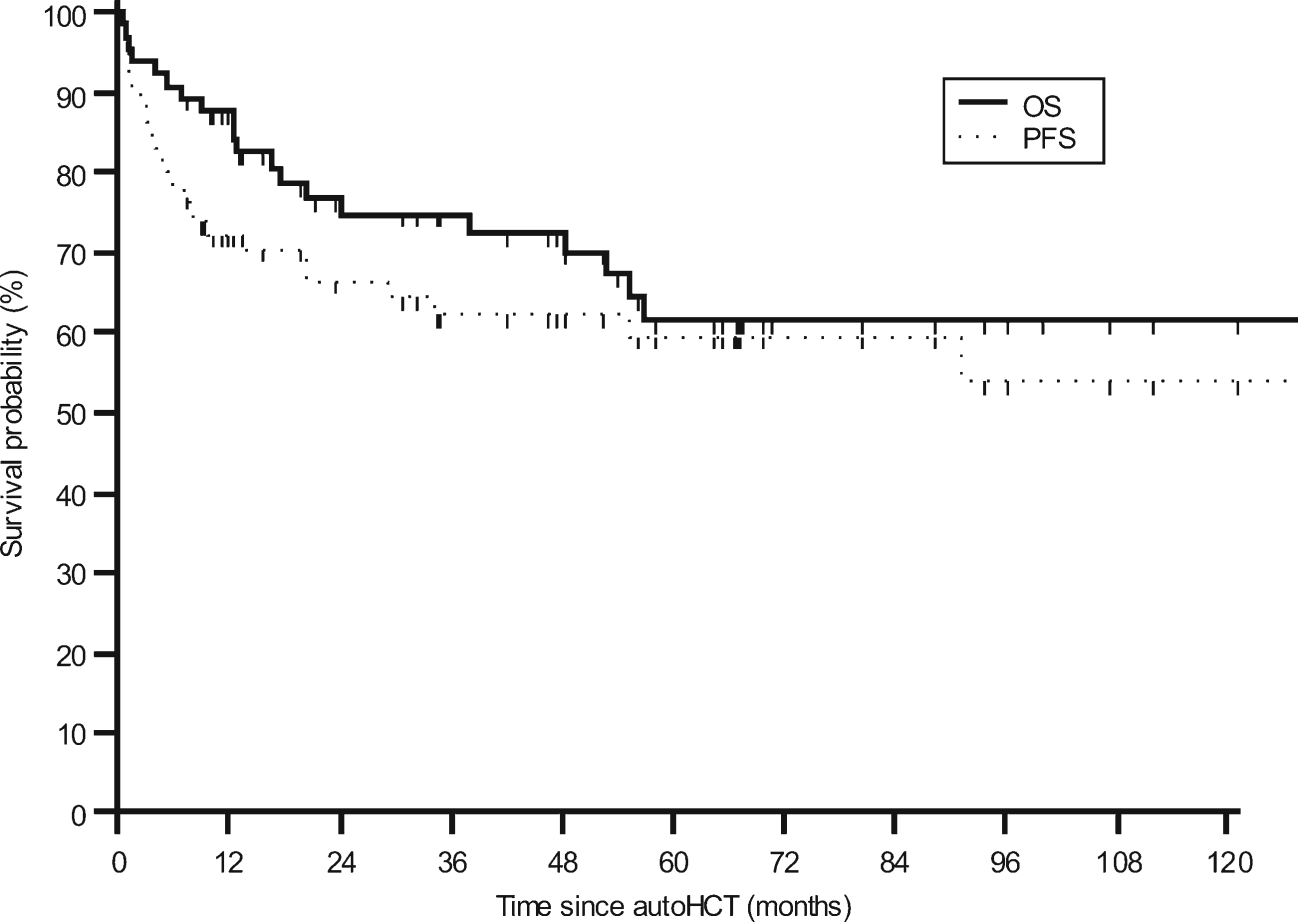
Kaplan–Meier estimates of overall survival (OS) and progression-free survival (PFS) for the whole group

At 3 and 5 years after transplantation, estimated OS for all patients was 74.7 % (95 % CI 62.2–84.1 %) and 61.5 % (95 % CI 47.0–74.2 %), respectively (Fig. 1). The respective PFS rates were 62.2 % (95 % CI 49.5–73.4 %) and 59.4 % (95 % CI 46.1–71.5 %) (Fig. 1). When patients were stratified by the response to the induction chemotherapy, the 5-year OS estimates were 66.0 % (95 % CI 49.9–79.1 %) and 41.0 % (95 % CI 16.6–70.9 %) for patients with PR/CR after first-line chemotherapy and with primary induction failure, respectively (*p* = 0.05). The corresponding 5-year PFS rates were 65.2 % (95 % CI 50.0–77.8 %) and 33.3 % (95 % CI 6.6–60.0 %) (*p* = 0.004).

Univariate analysis identified several risk factors for OS and PFS (Table 3). Two factors were found to be significant for OS: bone marrow involvement at diagnosis (*p* = 0.004) and response to induction chemotherapy (*p* = 0.050). Clinical stage at diagnosis and the presence of B symptoms tended to impact OS (*p* = 0.099 and 0.066, respectively). Poor PFS was associated with the presence of B symptoms at diagnosis (*p* = 0.023), bone marrow involvement at diagnosis (*p* = 0.002), and primary induction failure (*p* = 0.004). In multivariate analysis, bone marrow involvement at diagnosis and primary induction failure remained significant for both OS and PFS (Table 4).

**Table 3:** Univariate analysis of prognostic factors associated with overall survival and progression-free survival

Group	No.	5-year OS (95 % CI)	*p* value	5-year PFS (95 %)	*p* value
Ann Arbor stage					
I–II	14	84.6 (57.8–95.7)	0.099	78.6 (52.3–92.5)	0.10
III–IV	50	53.0 (36.5–68.8)		52.1 (36.8–67.0)	
B symptoms at diagnosis					
No	14	83.3 (55.1–95.3)	0.066	85.1 (58.8–95.8)	0.023
Yes	51	53.8 (37.3–69.5)		51.5 (36.6–66.2)	
Bone marrow involvement at diagnosis					
No	44	69.2 (50.9–82.9)	0.004	69.9 (53.7–82.3)	0.002
Yes	16	34.9 (15.1–61.9)		24.1 (8.8–51.1)	
Response to induction regimen					
≥PR	53	66.0 (49.9–79.1)	0.050	65.2 (50.0–77.8)	0.004
Primary refractoriness^a^	12	41.0 (16.6–70.9)		33.3 (6.6–60.0)	
Disease status at transplant					
CR	36	65.7 (46.9–80.6)	0.46	64.5 (46.3–79.3)	0.23
less than CR	29	57.1 (35.5–76.3)		54.7 (36.5–72.9)	
Number of prior chemotherapy regimen					
1	19	77.2 (53.7–90.7)	0.17	78.7 (56.6–90.8)	0.08
2 or more	46	56.8 (40.7–71.6)		51.7 (36.9–66.2)	
Histology					
ALK-positive and ALK-unknown ALCL	13	54.2 (26.2–79.8)	0.70	59.8 (33.4–81.5)	0.89
Other types	52	63.3 (47.1–77.0)		59.3 (44.4–72.6)	
ALCL-all subtypes	20	63.6 (38.8–82.8)	0.77	63.8 (41.7–81.3)	0.70
PTCL-NOS and AITL	45	60.7 (43.4–75.7)		57.5 (41.5–72.1)	
ALK-positive and ALK-unknown ALCL	13	54.2 (26.2–79.8)	0.74	59.8 (33.4–81.5)	0.62
ALK-negative ALCL	7	83.3 (43.7–97.0)		71.4 (35.8–91.8)	
PTCL-NOS	36	60.7 (42.3–76.5)		55.8 (39.0–71.3)	
AITL	9	55.6 (18.0–87.7)		55.6 (18.0–87.7)	

*OS* overall survival, *PFS* progression-free survival, *CI* confidence interval, *CR* complete response, *PR* partial response, *ALK* anaplastic lymphoma kinase, *ALCL* anaplastic large cell lymphoma, *PTCL* peripheral T cell lymphoma, *NOS* not otherwise specified, *AITL* angioimmunoblastic T cell lymphoma

^a^Less than PR

**Table 4:** Summary of results from overall survival and progression-free survival Cox model

Group	Overall survival		Progression-free survival	
	HR (95 % CI)	*p* value	HR (95 % CI)	*p* value
Response to induction chemotherapy				
Primary refractoriness^a^ vs ≥PR	3.21 (1.17–8.79)	0.023	4.56 (1.86–11.19)	0.001
Marrow involvement at diagnosis				
Yes vs no	3.85 (1.51–9.78)	0.005	3.58 (1.55–8.27)	0.003

*HR* hazards ratio, *CI* confidence interval, *CR* complete response, *PR* partial response

^a^Less than PR

In contrast, histology type (ALK-positive and ALK-unknown ALCL vs other types), LDH at diagnosis, extranodal involvement, mediastinal lymph nodes involvement, the number of pre-transplant chemotherapy lines, and the disease status at transplant (CR vs PR) were predictive for neither OS nor PFS (Table 3).

### Performance of risk models in prediction of outcome after autoHCT

**Fig. 2:**
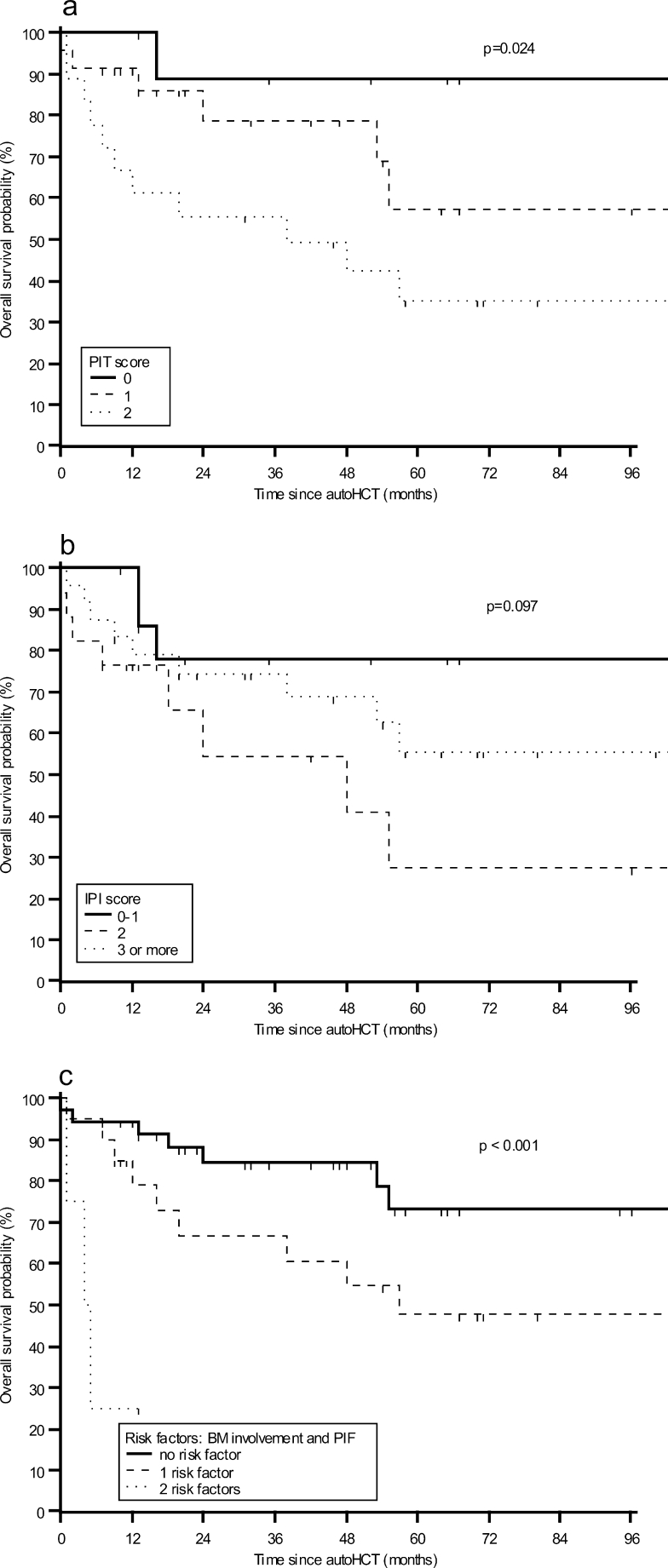
Kaplan–Meier estimates of overall survival (OS) **a** stratified by PIT score; **b** stratified by IPI score; and **c** stratified by the following risk factors: bone marrow (*BM*) involvement at diagnosis, primary induction failure (*PIF*)

Table 5 shows PIT and IPI performance in prediction of OS and PFS. Regarding PIT, only few studied patients were considered high-risk (score ≥3), so this small group was combined with intermediate–high-risk group (score 2). The 5-year OS was 89, 57, and 32 % for low (score 0), low–intermediate (score 1), and combined intermediate–high-risk and high-risk groups, respectively (*p* = 0.024, logrank test) (Fig. 2a). PIT score could reliably delineate score ≥2 risk group (HR 9.27; *p* = 0.033). However, PIT did not show statistical significant discrimination between patients with low- and low–intermediate-risk (HR 3.73; *p*, 0.22). Consequently, patients were divided into two groups according to a simplified two-class PIT. A simplified PIT provided a good discrimination between score 0–1 and score ≥2 risk group (HR 3.45; *p*, 0.011). In contrast to PIT, neither IPI nor a simplified two-class IPI was able to predict outcome after autoHCT (Fig. 2b).

**Table 5:** Performance of different scoring models in prediction of survival

Risk model	Risk group stratification	5-year OS (95 % CI)	HR (95 % CI)	*p* value	5-year PFS (95 % CI)	HR (95 % CI)	*p* value
IPI	Low (score 0–1)	77.9 (51.2–92.2)	1	0.12	74.5 (49.8–89.6)	1	0.23
	Int-1 (score 2)	27.3 (8.3–60.8)	3.90 (1.03–14.8)	0.046	28.5 (9.3–60.7)	2.76 (0.85–9.03)	0.09
	Int-2 and high (score 3–5)	53.1 (30.9–74.1)	2.16 (0.58–8.0)	0.25	51.1 (31.3–70.6)	2.29 (0.72–6.94)	0.17
PIT	Low (score 0)	88.9 (56.4–98.0)	1	0.036	90.0 (59.5–98.2)	1	0.059
	Low–int (score 1)	57.4 (30.4–80.6)	3.73 (0.45–31.13)	0.22	51.8 (28.6–74.3)	5.21 (0.66–41.31)	0.118
	Int and high (score 2–3)	31.8 (7.7–55.9)	9.27 (1.19–72.11)	0.033	32.4 (15.3–55.9)	9.52 (1.23–73.51)	0.031
Simplified two-class PIT	Score 0–1	70.0 (50.0–90.0)	1		65.4 (47.0–83.8)	1	
	Score 2–3	31.8 (7.7–55.9)	3.45 (1.33–8.91)	0.011	32.4 (15.3–55.9)	2.59 (1.12–6.01)	0.027

*OS* overall survival, *HR* hazards ratio, *IPI* the International Prognostic Index, *PIT* the Prognostic Index for peripheral T cell lymphoma, *Int* intermediate

Having found bone marrow involvement and primary induction failure as the only independent predictors of OS and PFS in the multivariate analysis, we classified the patients according to the number of identified independent unfavorable factors for outcome. The 2-year OS estimates were 72.1, 47.5, and 25 % for patients with zero, one, and two risk factors, respectively (*p* < 0.001) (Fig. 2c). The median overall survival was not reached for patients with none of the risk factors, compared to 57 months for patients with one risk factor and 4 months for patients with two risk factors. The respective 2-year PFS estimates were 73.4, 39.0, and 0 % (*p* < 0.001).

## Discussion

There are limited published prospective data regarding the outcomes of autologous stem cell transplantation in patients with peripheral T cell lymphoma. In addition, more extensive retrospective data are difficult to interpret. Most of the reported retrospective studies have been small and have included both patients with relapsed disease and those in first remission, as it has been pointed out in published reviews of the literature [21–23]. To overcome those limitations, we have included in the present analysis patients in first complete or partial remission after induction chemotherapy and patients with primary induction failure, who achieved at least partial remission after salvage treatment. Moreover, we have excluded patients with ALK-positive and ALK-unknown ALCL who were transplanted in first complete remission achieved with an initial induction chemotherapy to make the results more clear for interpretation, as ALK-positive ALCL is known to carry a better outcome comparing to other histological subtypes of PTCL. Among the 65 patients in this report, 36 of them underwent autoHCT in complete remission, whereas 29 patients were in partial remission at transplant. The 5-year OS for all patients was 61.5 %, what is consistent with previous reports of prospective and retrospective studies, across which the range of OS was from 48 to 68 % at 4–5 years for patients transplanted in first remission [10, 11, 18, 24, 25]. We did not find a statistically significant survival difference by histological subtype of PTCL. Similarly to our results, some previous studies also showed no difference in survival after autoHCT based on histology [11, 25]. In addition, our study did not show that disease status at transplant (CR vs PR) significantly affected survival after autoHCT. However, it should be pointed out that the difference in survival based on disease status at transplant was found mostly in the studies including patients with both chemosensitive and chemorefractory disease at transplant [11, 19, 25]. Moreover, those studies included both patients in first remission and patients in second remission achieved after previous relapse. In contrast to disease status at transplant, we identified B symptoms at diagnosis, bone marrow involvement at diagnosis, and refractoriness to induction chemotherapy as risk factors adversely influencing overall survival and progression-free survival after autoHCT in univariate model. In multivariate analysis, bone marrow involvement at diagnosis and response to induction chemotherapy remained the only independent prognostic factors associated with OS and PFS. Our results based on limited number of patients suggest that the transplant outcomes for patients with bone marrow involvement at diagnosis and primary refractoriness to induction chemotherapy are very poor with 25 % alive at 5 years, in contrast to 47 and 72 % survivors among patients with one risk factor and without any of those independent risk factors, respectively. Although the group of patients with two risk factors was very small, consisting of only four patients, it is worth pointing out that all of those patients experienced disease progression within 8 months of autoHCT, and three of them died from that reason.

We also evaluated the predictive value of the IPI and PIT scoring systems for transplant outcome in patients with PTCL. The prospective trials have shown conflicting results regarding the role of the IPI index. The IPI score did not provide a good discrimination between the risk groups prior to autoHCT in the current analysis, which is in line with the results of the both German and Spanish prospective multicenter trials [8, 18], but in contrast to the results of the Italian prospective study [26]. The PIT score initially proposed by Gallamini and coworkers for patients with PTCL-NOS was previously reported to be predictive for transplant outcome [8, 18, 19]. Although this model reliably delineated the combined intermediate–high- and high-risk group in the current analysis, it failed to discriminate well between low- and low–intermediate-risk categories. However, a simplified PIT provided a good discrimination between score 0–1 and score ≥2 risk group (*p* = 0.011), with 70 % of patients alive at 5 years in low-risk group, in contrast to 32 % in high-risk group.

In conclusion, the treatment strategy for the patients with bone marrow involvement at diagnosis, who did not respond to induction chemotherapy, as well as for patients with PIT score ≥2 at diagnosis remains an area for further studies with newer treatment options, including RIC allogeneic HCT, since the results of HDT followed by autoHCT are poor in this setting. Despite the limitation of the retrospective study, like no intent-to-treat analysis, our results support the usefulness of the PIT in stratifying PTCL patients undergoing autoHCT in first remission and expanding the published experience may be useful in patient selection for autotransplant.
